# SPD-CNN: A plain CNN-based model using the symmetric positive definite matrices for cross-subject EEG classification with meta-transfer-learning

**DOI:** 10.3389/fnbot.2022.958052

**Published:** 2022-08-03

**Authors:** Lezhi Chen, Zhuliang Yu, Jian Yang

**Affiliations:** ^1^College of Automation Science and Engineering, South China University of Technology, Guangzhou, China; ^2^Pazhou Laboratory, Guangzhou, China

**Keywords:** EEG, Motor imagery, SPD matrices, CNN, Meta-transfer-learning

## Abstract

The electroencephalography (EEG) signals are easily contaminated by various artifacts and noise, which induces a domain shift in each subject and significant pattern variability among different subjects. Therefore, it hinders the improvement of EEG classification accuracy in the cross-subject learning scenario. Convolutional neural networks (CNNs) have been extensively applied to EEG-based Brain-Computer Interfaces (BCIs) by virtue of the capability of performing automatic feature extraction and classification. However, they have been mainly applied to the within-subject classification which would consume lots of time for training and calibration. Thus, it limits the further applications of CNNs in BCIs. In order to build a robust classification algorithm for a calibration-less BCI system, we propose an end-to-end model that transforms the EEG signals into symmetric positive definite (SPD) matrices and captures the features of SPD matrices by using a CNN. To avoid the time-consuming calibration and ensure the application of the proposed model, we use the meta-transfer-learning (MTL) method to learn the essential features from different subjects. We validate our model by making extensive experiments on three public motor-imagery datasets. The experimental results demonstrate the effectiveness of our proposed method in the cross-subject learning scenario.

## 1. Introduction

An EEG-based Brain-Computer Interface (BCI) is a system to measure and analyze the electroencephalography (EEG) brain signal (Rao, 2013), thus enabling the communication or interaction between the brain and external environment (Kothe and Makeig, 2013). Recent research has opened up the possibility for EEG signals to apply in rehabilitation (Tariq et al., 2018), entertainment (Nijholt et al., 2008), and transportation (Göhring et al., 2013) because of the harmless, non-invasive, and inexpensive features of the EEG-BCI. Motor imagery (MI), which refers to the mental simulation of body movements, is a famous paradigm of the EEG-BCI system (Lotze and Halsband, 2006). MI signals are widely used in the BCI system (Alamgir et al., 2010; Arvaneh et al., 2013; Jayaram et al., 2016) because of their flexibility in reflecting the bioelectrical activity of the brain. These signals attract increasing attention in rehabilitation therapy (Naseer and Hong, 2013, 2015; Hong et al., 2015).

However, due to the separation between the signal source (inside the human brain) and the detector, the EEG signals would be easily contaminated by various artifacts and noise, including muscle movements, eye blinks, heartbeats, and environmental electro-magnetic field in the applications of the BCI-system. This phenomenon induces a domain shift in each subject, even in different sessions of the same subject (Reuderink et al., 2011), and exhibits significant pattern variability between different subjects. Consequently, it hinders people from using the data generated from different subjects to improve the performance of the BCI system (Lotte and Guan, 2010) and increasingly reduces the accuracy and stability of EEG cross-subject classification. Currently, the users of the BCI-system have to provide tons of EEG-data to build a user-specific classifier so that the system can work properly. Accordingly, it greatly lengthens the time of calibration of the BCI system and heavily inhibits BCI-system development.

To overcome this problem, lots of methods are proposed to eliminate the shifting problem of data distribution among different subjects. Rodrigues et al. (2018) present a transfer Learning approach to match the statistical distributions of different sessions/subjects. This method allows the BCI systems to reuse the data from different users and reduce the calibration time. He and Wu (2019) propose a Euclidean Space Data Alignment Approach to align the time-domain EEG trials in the Euclidean Space and alleviate the domain shift between different sessions and subjects successfully. However, this kind of **data-augmentation** method normally classifies the data by the traditional geometry-aware classifiers (such as support vector machine and the minimum distance to mean classifier) (Barachant et al., 2013), which are insufficient for feature extraction. Also, it requires people to use certain prior knowledge of brain science.

With the development of machine learning, deep learning technology has been applied to extract discriminative features from EEG (Lotte et al., 2018) and many **model-based** learning algorithms have been proposed for MI-EEG cross-session/subject classification (Wu et al., 2020). Schirrmeister et al. (2017) focus on the application of different CNN architectures in EEG-MI classification and design an efficient network architecture to decode information from the EEG-MI signal. This method shows the powerful feature extraction ability of CNN and draws a great deal of attention to the applications of CNN in the BCI system. Lawhern et al. (2018) propose a brand-new compact CNN-based model called EEGNet, which contains depth-wise and separable convolutions to extract the descriptive information from EEG signal directly. This network structure is robust enough to learn a wide variety of interpretable features over a range of BCI tasks in cross-session/subject learning and gain outstanding classification performance. Fahimi et al. (2019) propose an inter-subject transfer learning framework built on top of the CNN model which is fed into three different EEG representations and transfers knowledge between different subjects thus avoiding time-consuming re-training. However, this kind of network focus on the feature extraction of EEG signal and their performances would deteriorate when the data of the user are insufficient, especially in the few-shot scenario of cross-subject learning.

In the most recent studies, meta-learning, which is a task-level learning method, has seen substantial advancements in computer vision and speech recognition recently (Vanschoren, 2018). This kind of learning method helps the neural network to extract usable features from related tasks and largely increases the generalization ability of the neural network. Li et al. (2021) use the training method called Model-Agnostic Meta-Learning (MAML) (Finn et al., 2017) and build the CNN-based classifier which combines one and two dimensional-CNN layers to improve the accuracy of the MI-EEG classification. However, these kinds of meta-learning structure are very sensitive to neural network architectures (usually shallow neural networks), which often leads to instability during training and easily induces overfitting problems. Therefore, it limits the effectiveness of meta-learning.

In consequence, given the above, an effective model that is capable of capturing essential features and a robust meta-learning method are both essential to cross-subject learning in EEG classification. The symmetric positive definite (SPD) matrices have been widely used in motor imagery EEG-based classification over the past few decades (Barachant et al., 2013; Xu et al., 2021), because of their capacity to capture informative structure from the data (Huang and Van Gool, 2017). In terms of the ability to capture input data structure, the CNN has the powerful capability of extracting features of two-dimensional matrix-shape data (LeCun et al., 1998; Krizhevsky et al., 2012) and the SPD matrices are one of the two-dimensional matrix-shape data. Therefore, Hajinoroozi et al. (2017) combine the SPD matrices of EEG data and the deep learning method and present a series of deep covariance learning models for drivers' fatigue prediction, which explore the potential of this kind of method for the application of BCI system. Inspired by this, we propose a plain CNN-based model called SPD-CNN, which transforms the EEG signal into the SPD matrices and uses a CNN with five convolutional layers to capture the features of SPD matrices. Also, we apply a cutting edge meta-updating strategy called the meta-transfer-learning (MTL) (Sun et al., 2019) which combines the advantage of transfer learning and meta-learning to extract the subject invariant features and alleviates the shifting problem between the source domain (training subjects) and the target domain (test subjects). The major contributions of this article can be summarized as follows.

- The SPD-CNN model we proposed uses the SPD matrices of the EEG signal as descriptors to highlight the spatial information of the EEG-MI signal and reduces the diversity of EEG data characteristics of different subjects. Additionally, the proposed descriptor tremendously decreases the size of data and effectively reduces the difficulty of feature extraction.
- Using the MTL as our learning strategy helps the network extract the crucial features. In other words, it can transfer the domain knowledge between different subjects during the training process and enhance the robustness of the network in the BCI system.
- To the best of our knowledge, the network we proposed is simple to design and has fewer parameters than most networks for EEG classification currently. Therefore, it could simplify the training process tremendously and shortens the training time extremely.

The remainder of the article is organized as follows. Section 2 presents the framework of the proposed approach. Section 3 describes the experimental settings, then shows the results, and provides a comprehensive analysis. The effectiveness of the proposed SPD descriptor is discussed in Section 4 and the conclusion is summarized in Section 5.

## 2. Materials and methodology

### 2.1. Data description

We present examples with three public EEG-MI datasets which are BNCI2014001 (Tangermann et al., 2012), BNCI2015004 (Scherer et al., 2015), and Sch2017 (Schirrmeister et al., 2017).

BNCI2014001 consists of the EEG data from 9 subjects and this MI-paradigm consists of four different motor imagery tasks that the subjects are required to make the imagination of movement of the left hand, right hand, both feet and tongue. The EEG Signals are recorded with 22 electrodes at a 250 Hz sampling rate and two sessions were recorded for each subject. Each session is composed of 6 runs separated by short breaks. One run consists of 48 trials (12 for each of the four possible classes), yielding a total of 288 trials per session.

BNCI2015004 is a 30-electrode dataset obtained from 14 subjects with disability (spinal cord injury and stroke). The dataset consists of five classes of imagined movements of right-hand and feet, mental word association, mental subtraction, and spatial navigation. The EEG signals are recorded at a 250 Hz sampling rate, and two sessions were recorded for each subject. Each session consists of 8 runs, resulting in 40 trials of each class. The EEG signals were bandpass filtered 0.5–100 Hz and sampled at a rate of 256 Hz.

Sch2017 is a 128-electrode dataset obtained from 14 healthy subjects [6 women, 2 left-handed, age 27.2 ± 3.6 (mean ± std)] and this MI-paradigm consists of four different motor imagery tasks which ask subjects to make the imagination of movement of the left hand, right hand, both feet, and rest (no movement), with roughly 1,000 four-second trials of executed movements divided into 13 runs (each run consist of the approximately 1,000 trails per subject.

Three datasets mentioned above are publicly available on the "Mother of all BCI Benchmarks"(MOABB) framework (Jayaram and Barachant, 2018). In the experiment section, the subjects in the same dataset will be divided into training subjects, validation subjects, and test subjects who provide data for the training set, validation set, and test set for the cross-subject learning experiments, respectively. More details can be seen in Table 1.

**Table 1:** Key information of the three MI-EEG datasets used for experiences.

**Dataset**	**Number**	**Trails**	**Class**	**Band pass**	**Number of**
	**of subjects**	**per subject**		**filter (Hz)**	**electrodes channnels**
BNCI2014001	9	576	4	4–250	22
BNCI2015004	9	400	5	0.5–100	30
Sch2017	14	1,000	4	4–250	128

### 2.2. SPD-CNN model

Table 2 gives a brief description of the mathematical symbols that will be used in the rest of the article.

**Table 2:** Table of symbols used in the article.

**Symbols**	**Meaning**
*F*(Θ, θ)	The classification network with parameter Θ and θ
Θ, θ	The parameter of the feature extractor block and the classifier block
Θ^*pre*^, Θ^*meta*^	The parameter after the pre-train phase and the meta-adaption phase
*f*(Θ, θ)	The network after the *F*(Θ, θ) is upgraded by specific task
*X*	The multiple time-series of a EEG matrix
*C*	The covariance matrix estimated by *X*
*D*_*tr*_, *D*_*val*_, *D*_*te*_	Dataset *D* for Training,Validation and Testing phase
*T*_*i*_	The specific task which is sample from the *D*
*l*_*i*_	The loss function in task i during the inner-loop
*L*_ϕ_	The meta loss function in meta training

As mentioned above, we are particularly interested in the case where the SPD matrices are spatial covariance matrices, which describe the second-order statistics of zero-mean multivariate time series. We assume that the information on the power and spatial distribution of EEG sources can be coded by a covariance matrix. Therefore, the spatial covariance matrix *C* of a T-sample realization of a zero-mean *d*-dimensional time series (*d* being the number of electrode channels) *X* ∈ *R*^^*d*^^×^*T*^, is estimated as


(1) $$\begin{array}{l} C_{i}=\frac{1}{T}X_{i}^{T}X_{i},i=1,2,\ldots ,n \end{array}$$

where *X*_*i*_ is the sample from the EEG dataset *D* = {*X*_*i*_, *i* = 1, 2, ⋯ , *n*} and n is the total amount of samples in dataset *D*.

Based on the analysis above, we develop a covariance matrix estimator called the SPD descriptor that captures not only the diversity among different electrode channels but also the statistical properties of EEG image regions. The descriptor is capable of estimating the *d*×*d* covariance matrix of the EEG features mentioned in Equation (1). Then, these matrices are normalized with the whole sample set mentioned in Equation (2) to improve the numerical stability of the model. Consequently, the network is able to focus on the critical features and accelerate the learning process (Shanker et al., 1996).


(2) $$\begin{array}{l} C_{i}^{*}=(C_{i}-C_{mean})/C_{std},i=1,2,\cdots ,n \end{array}$$

where *C*_*mean*_, *C*_*std*_ is the mean and SD of covariance matrix set *C* = {*C*_*i*_, *i* = 1, 2, ⋯ , *n*} and $C_{i}^{*}$ is the output sample of the descriptor.

**Figure 1:**
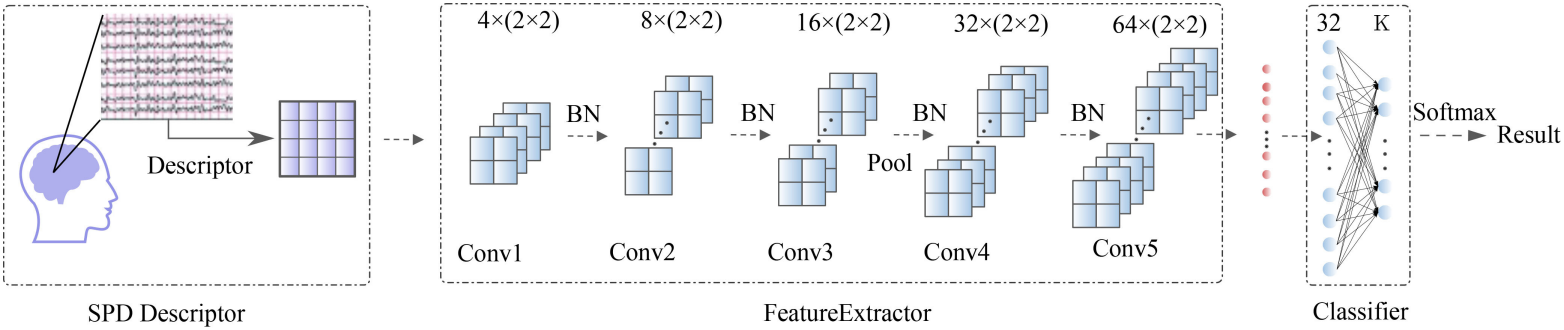
Overall visualization of the SPD-CNN architecture. It starts with an SPD descriptor to transform EEG into SPD matrices, then the matrices are encoded by Feature Extractor Block and flattened as the input data of the classification block. In the classification block, the features are passed to a two-fully connected layer and put into a soft-max classification with K units, K is the number of classes in the data.

After being processed by the SPD descriptor, the *d*×*d* matrices are taken into a Feature Extractor block. This block contains five convolutional layers (*Conv*1, *Conv*2, *Conv*3,*Conv*4, *Conv*5) with minimum convolution kernel (2 × 2). Then the output data from Feature Extractor were flattened and taken through a classifier block with a two-layer fully-connected network (*FC*) onto the BCI outputs. A whole visualization and full description of the SPD-CNN model can be found in Figure 1 and Table 3.

**Table 3:** Basic parameter of SPD-CNN model.

**Block**	**Layers**	**Size and Kernel**	**Activation**
Feature extractor	*Conv*1	4 × (2 ×2)	Relu
	*Conv*2	8 × (2 ×2)	Relu
	*Conv*3	16 × (2 ×2)	Relu
	*Max*−*pool*	(2 ×2)	-
	*Conv*4	32 × (2 ×2)	Relu
	*Conv*5	64 × (2 ×2)	Relu
Clasiifier	*FC*	2 ×32	softmax

In the Feature Extractor block,Conv means a convolutional layer and FC represents a two-layer fully-connected network, with 32 neurons inside the hidden layer as shown in Figure 1.

### 2.3. Training structure and learning strategy

Our training structure is to help the model extract the key features through learning a better initial set of parameters from various tasks of different subjects. Hence, the network gains a fast adaption to new user tasks using only a few data. This learning strategy is based on the assumption that the EEG data from different subjects share the same representative features. These features are just masked by the effect of individual variation and wide discrepancy in the experiment environment. In this section, we illustrate the main idea of MTL and describe its application in the EEG cross-subject learning scenario.

**Figure 2:**
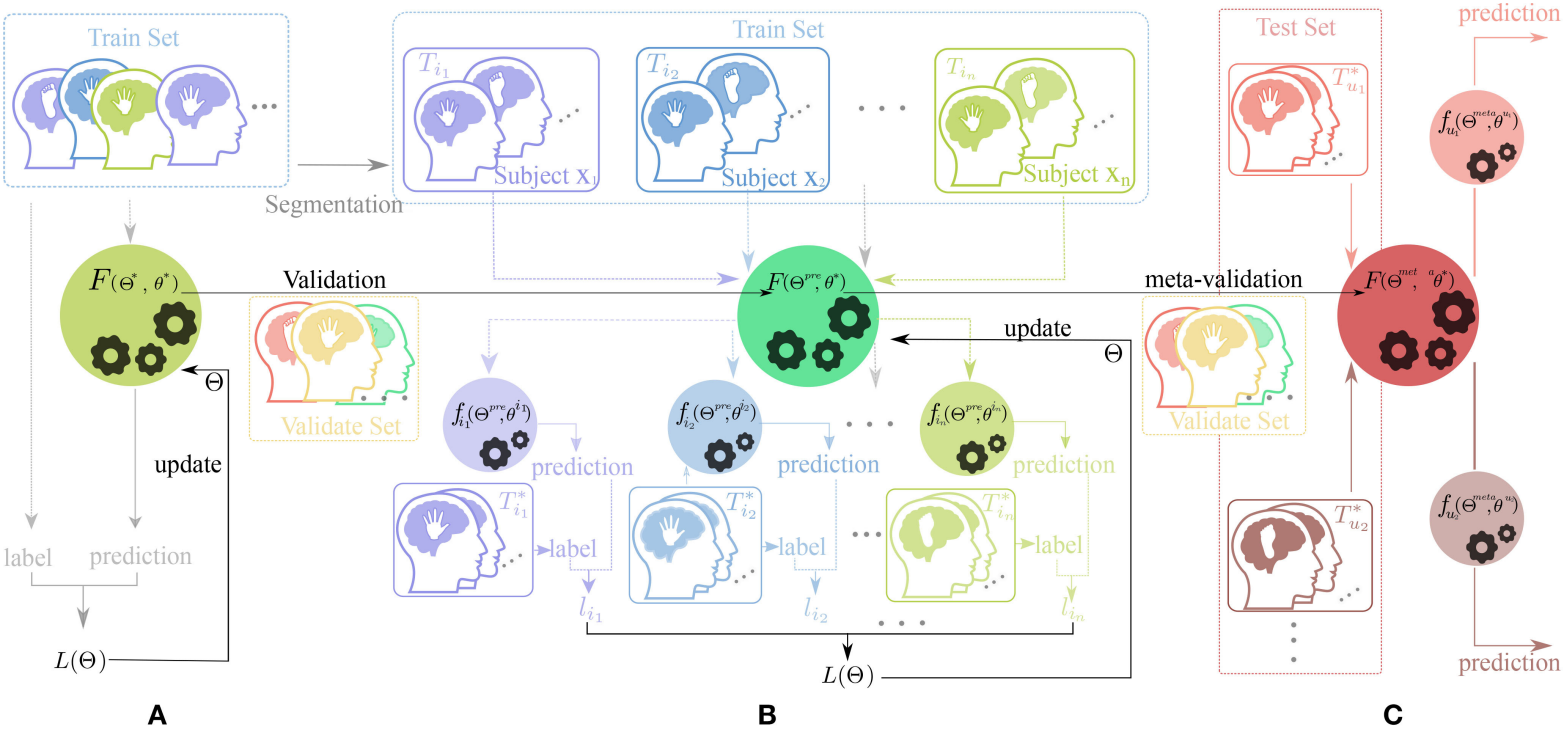
Workflow of our training framework. The dataset for training, validation, and test process is displayed on different rectangular regions with the colors blue, yellow, and red, respectfully. The picture of human heads in different colors (such as purple, blue, yellow, brown, and red) with a hand or feet inside represent the data from different subjects doing motor-imagery tasks. The black horizontal lines with a black arrow represent the change of the parameter of the neural network and the colorful vertical and horizontal lines (such as purple, blue, gray, and green) indicate the direction of data flow. In addition, the black gears in the circle represent the update process of parameters. **(A)** Pre-train phase, **(B)** Meta-update phase, and **(C)** Domain-adaption phase.

The MTL combines the advantage of transfer learning and meta-learning structure. This training method uses the fine-tune skill and model-agnostic meta-learning (MAML) algorithm (Finn et al., 2017) with a novel constrained setting on network parameters called scaling and shifting (SS) operation to solve the overfitting problem. Hence, our training framework consists of three parts: Pre-train, Meta-updating, and Fast adaption. The whole workflow in this framework is shown in Figure 2.

**Figure 3:**
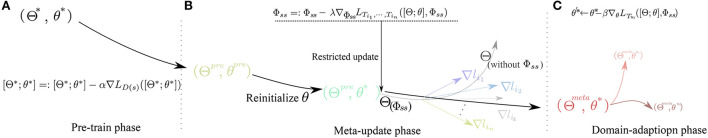
Diagram of parameters variation through the learning process in different phases. **(A)** Pre-train phase, **(B)** Meta-update phase, and **(C)** Domain-adaption phase.

As shown in Figures 2, 3, in the Pre-train phase, data of training subjects are merged randomly into a training dataset *D*_*tr*_ for classifier *F*(Θ^*^, θ^*^). The network *F* with initialized parameter (Θ^*^, θ^*^) is optimized by the traditional gradient descent method (refer to Equation 3) and gains the better initialized parameter(Θ^*pre*^, θ^*pre*^).


(3) $$\begin{array}{l} \left[\Theta^{*};\theta^{*}\right]=:\left[\Theta^{*};\theta^{*}\right]-\alpha \nabla L_{D_{tr}}\left(\left[\Theta^{*};\theta^{*}\right]\right) \end{array}$$

where α is the learning rate of and *L*_*D*_*tr*__ denotes the most frequently used empirical loss in machine learning like cross-entropy (Zhang and Sabuncu, 2018). This process neglects the domain shift from different subjects and provides a rough direction for the network to upgrade the parameter.

In the meta-update phase(b), we randomly re-initialize the parameter θ^*^ first and use the MAML structure (Finn et al., 2017) as a training structure with constraining parameter Φ_*ss*_. The Φ_*ss*_ is updated by Equation (4) as follows,


(4) $$\begin{array}{l} \Phi_{ss}=:\Phi_{ss}-\lambda \nabla_{\Phi_{ss}}L_{T_{i_{1}},\cdots \;,T_{i_{n}}}\left(\left[\Theta ;\theta \right],\Phi_{ss}\right) \end{array}$$

where λ is the learning rate during the update process of Φ_*ss*_. The main idea of constraining parameter Φ_*ss*_ is to restrict the learning process of weight and bias in each convolutional layer, which means the weights and the biases of the same CNN layer are scaled and shifted as a whole, respectively.

To be specific, the weights *W* in the same CNN layer will time a scaling factor Φ_*s*_1__ and the biases *b* in the same CNN layer will add a shifting factor Φ_*s*_2__ through an update process. Assuming X is the input data, the SS operation could be expressed by Equation (5).


(5) $$\begin{array}{l} SS\left(X;,W,b;\Phi_{s_{1}},\Phi_{s_{2}}\right)=\left(W⊙\Phi_{s_{1}}\right)X+\left(b+\Phi_{s_{2}}\right) \end{array}$$

where ⊙ denotes the element-wise multiplication (For details, refer to the article by Sun et al., 2019).

Inside the MAML learning framework, we sample the data of *j* classes (where *j* is the number of ways in few-shot learning) from the same training subject for a task. Therefore, each subject-specific task is seen as an independent sample of the same classification problem.

More specifically, the train set *D*_*tr*_ was segmented into different training tasks *T*_*i*_*k*__ and test tasks $T_{i_{k}}^{*}$,where *T*_*i*_*k*__⊂*T* = {*T*_*i*_1__, *T*_*i*_2__, ...*Ti*_*n*_} and $T_{i_{k}}^{*}\subset T^{*}=\left\{T_{i_{1}}^{*},T_{i_{2}}^{*},...Ti_{n}^{*}\right\}$, n being the number of tasks in meta-learning. Significantly, the data of *T*_*i*_*k*__ and $T_{i_{k}}^{*}$ are sampled from the same training subject *x*_*i*_ and the data of the subject-specified task (*T*_*i*_*k*__ or $T_{i_{k}}^{*}$) are divided into training data and test data for the training process. As a result, the generalized model *F*(Θ^*pre*^, θ^*^).

will be trained into different subject-specified networks $f\left(\Theta^{pre},\theta^{i_{k}}\right)$ by gradient descent method. Also, after training the $f_{i_{k}}\left(\Theta^{pre},\theta^{i_{k}}\right)$ with the training data of the test task $T_{i_{k}}^{*}$ again and calculating the loss function based on the test data of the $T_{i_{k}}^{*}$, each network $f\left(\Theta^{pre},\theta^{i_{k}}\right)$ would generate subject-specified loss *l*_*i*_*k*__. After updating the parameter Θ^*pre*^ several learning epochs, which is guided by the meta-loss *L*(Θ) based on different subject-specified loss *l*_*i*_*k*__(refer to Equation 6), the parameters Θ^*meta*^ with better generalization ability are selected by validate set *D*_*val*_ through the meta-validation process.


(6) $$\begin{array}{l} L{\left(\Theta \right)}_{T}=\sum l_{i_{k}},k=1,2,\cdots \;,n \end{array}$$

In the domain-adaption phase(c), we fix the parameter of Feature Extractor Θ^*meta*^ learned from the Meta-update phase and use the Fine-tune skill to train a user-specify network $f\left(\Theta^{meta},\theta^{u_{j}}\right)$, which is greatly adapted to the user *u*_*j*_ pattern. In this process, a few data of the user from the test set are used to train the *F*(Θ^*meta*^, θ^*^) into $f\left(\Theta^{meta},\theta^{u_{j}}\right)$ and the parameter of the classifier block is updated by the Equation (7).


(7) $$\begin{array}{l} \theta^{{*}^{\prime }}\leftarrow \theta -\beta \nabla_{\theta }L_{T_{u_{j}}}\left(\left[\Theta^{meta};\theta^{*}\right],\Phi_{ss}\right) \end{array}$$

where β is the learning rate during the update process. After this phase, the network is greatly adapted to the situation of user *u*_*j*_ and gains better prediction in the BCI system.

## 3. Experiments and results

Our experiments aim to assess whether SPD-CNN is capable of extracting the discriminative information of EEG data recorded from different subjects and evaluate the transfer capacity of our proposed learning structure in the cross-subject scenario based on the recognition accuracy in the few-shot learning framework.

### 3.1. Implementions details

We conduct normal machine learning and few-shot learning experiments on the cross-subject scenario. In these experiments, we compare SPD-CNN with two wildly used models, DeepConvNet (Lawhern et al., 2018) and EEGnet (Schirrmeister et al., 2017), which perform well on EEG classification with code publicly available. The experiments show the different performance of classification between our training strategy and the benchmark of transfer learning methods in EEG classification.

In the experiences of datasets BNCI2014001 and BNCI20150004, we choose three subjects for the validation set, two subjects as the user for the test set and all the remaining subjects for the training set randomly. This choosing process repeats 18 times, thus, producing 18 different folds. We follow the same procedure for the experiences of dataset Sch2017 except we increase the number of validate subjects to 5 and generate 28 folds.

In the few-shot scenario, we consider the 4-class classification (5-class classification for BNCI2015004), so we sample 4-class(5-class classification for BNCI2015004), 5-shot/10 shot episodes to contain 5 or 10 samples for a train episode and 10 samples (each class) for episode test.

The parameter of the network in our experiments are initialized by the normalization method from He et al. (2015) and the whole model is trained by Adam optimizer (Kingma and Ba, 2014). The learning rates α, λ, and β of all learning phases are initialized as 0.001 and dropped by 1% every 10 epochs. All the loss functions are the normal form of cross-entropy cause there is no sample imbalance problem in all datasets (Fatourechi et al., 2008). In the Pre-train phase, the batch size is set to 64 and the network will be trained 50 epochs in each fold. In the experiments of MTL, each task is sampled from the same subjects of all classes evenly in the meta-update phase. Furthermore, we use 60 tasks that form 12 meta-batch(5 tasks for each meta-batch) in one training update loop and choose 30 random tasks for meta-validation and meta-test. In the meta-update phase, the network will be trained 40 epochs in each fold.

All the models were implemented based on PyTorch (Paszke et al., 2019) and trained on a single GPU of 12 GB TITAN-Xp with Intel Xeon CPU E5-2620 v4 as CPU. More details can be found in the GitHub repository https://github.com/sabinechen/SPD-CNN-Using-Meta-Transfer-Learing-EEG-Cross-Subject-learning.

### 3.2. Experimental evaluation

**Figure 4:**
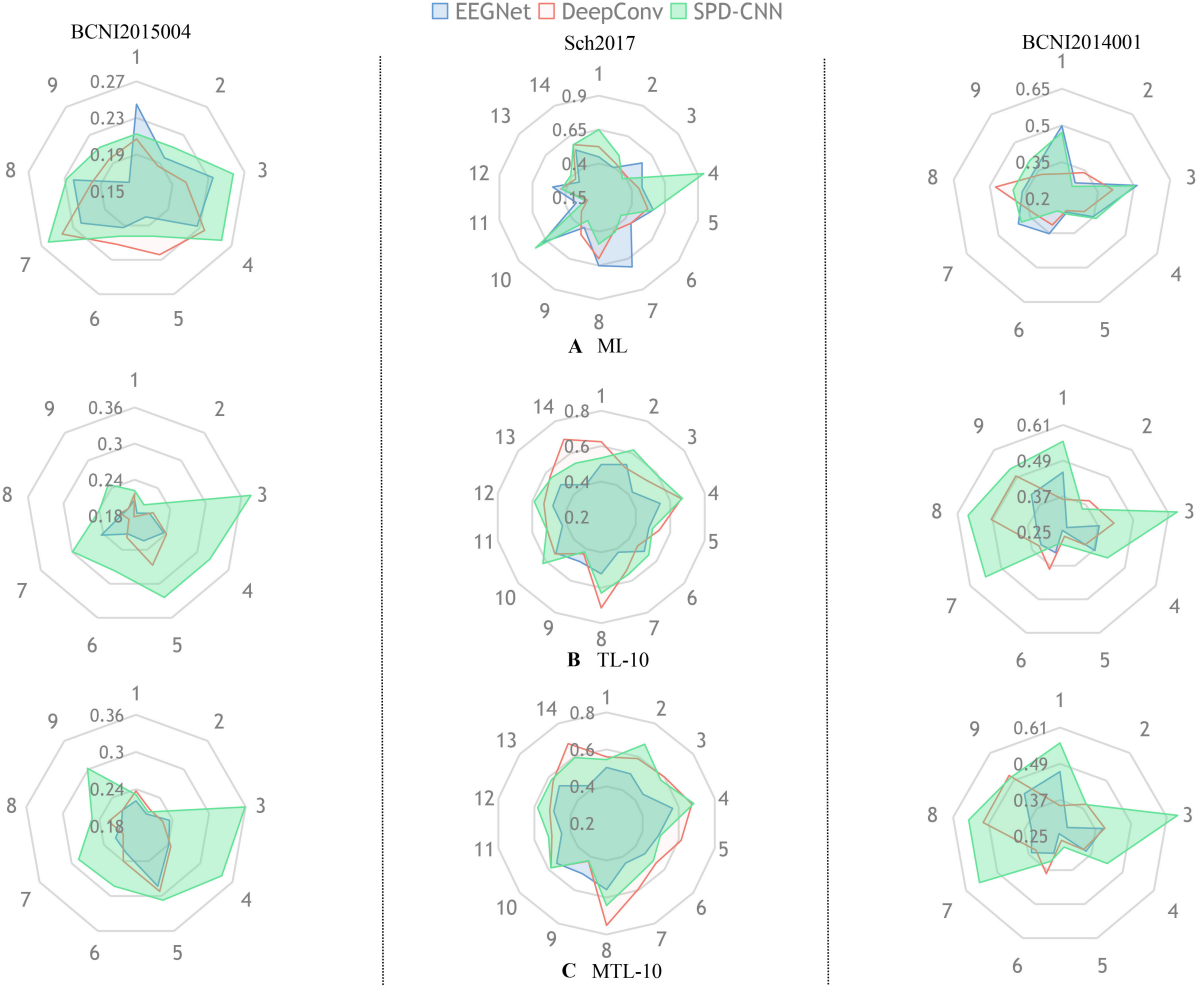
In each radar picture, every axis is assigned a variable that represents the classification accuracy of the specific subject (such as 1:subject1 and 2:subject2) and the different colors represent different network architectures (Blue:EEGNet,Red:DeepConvNet,Green:SPD-CNN).Also, the radar pictures arranged in the same column are shown the performance of experiences in the same dataset. The subgraph **(A)** represents the experiments that train the network with the ML method using zero-shot in the target domain. The subgraphs **(B,C)** represent the experiments that train the network with TL and MTL methods, respectively and fine-tune the network using 10shot on the target domain.

**Figure 5:**
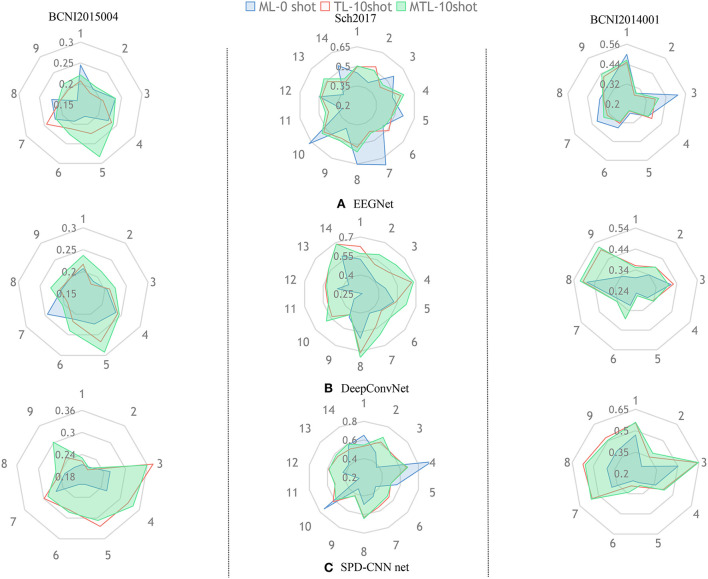
The aim of this radar picture is to show the different performances using different training methods and different colors represent different training strategies (Blue:ML,Red:TL,Green:MTL).The three subgraphs **(A–C)** represent the classification performance of the three models, respectively.

To show the effectiveness of our model and learning strategy, we design some comparative experiments and ablative settings: Three networks are trained on the chosen dataset using Normal Machine Learning (ML), Transfer Learning (TL), and MTL method. In the experiments of ML, we train the networks from scratch only using the source-domain data, which is also called zero-shot.In the experiments of TL, we pre-train the networks on the source domain and fine-tune the classifier block of the networks on the target domain (5-shot and 10-shot). Figures 4, 5 provide a qualitative summary of the results for the cross-subject classification accuracy.

Figure 4 gives an overall picture of the performances obtained by training EEGNet, DeepConvNet, and SPD-CNN net on the target domains (10shot) with three learning strategies: ML, TL, and MTL. It shows that the three networks show noticeably varying patterns in the accuracy of different subjects in cross-subject learning. The green area, which represents the performance of SPD-CNN, almost covers other different color areas. It reveals that SPD-CNN has the remarkable ability to transfer source domains (train set) to the target domain (test set) in three datasets.

Figure 5 gives an overall picture of the performances of using different learning strategies on different networks. It shows that the coverage area of MTL is more evenly distributed in all dimensions than other learning strategies in most cases, indicating that the MTL strategy performs better than the other two learning strategies in the experiments. Therefore, we can conclude that the MTL learning strategy strengthens the generalization ability and robustness of the networks.

Furthermore, we present the accuracy of different experiments and give a quantitative summary of the results in Table 4 below.

**Table 4:** The 4-way, 10-shot, and 5-shot classification accuracy (%) on three datasets (5-way for BNCI2015004).

**Dataset**	**BNCI2014001**				
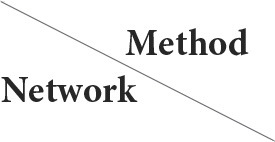	**ML**	**TL-10**	**MTL-10**	**TL-5**	**MTL-5**
	
EEGNet	**37.65 ± 2.847**	35.25 ± 2.7	35.95 ± 3.56	28.68 ± 3.17	29.84 ± 3.15
DeepConv	33.96 ± 3.14	38.75 ± 4.32	38.8 ± 3.9	34.52 ± 3.52	35.64 ± 3.4
**SPD-CNN**	36.88 ± 3.56	**46.78 ± 2.78**	**47.44 ± 4.1**	**42.99 ± 2.78**	**43.39 ± 2.63**
	**BNCI2015004**				
EEGNet	20.88 ± 2.72	21.22 ± 3.32	22.76 ± 2.36	21.3 ± 4.28	22.79 ± 3.4
DeepConv	20.46 ± 2.23	21.37 ± 2.21	23.29 ± 3.02	21.74 ± 3.76	22.47 ± 4.31
**SPD-CNN**	**22.74 ± 2.45**	**27.92 ± 3.3**	**28.57 ± 2.58**	**25.62 ± 3.63**	**26.82 ± 3.77**
	**Sch2017**				
EEGNet	50.2 ± 4.23	48.07 ± 3.91	49.27 ± 3.12	43.82 ± 4.21	45.13 ± 4.26
DeepConv	44.25 ± 3.06	56.02 ± 3.49	**59.22 ± 4.39**	51 ± 2.67	**56.4 ± 3.53**
**SPD-CNN**	**50.44 ± 3.04**	**56.31 ± 4.53**	56.92 ± 3.79	**51.13 ± 3.44**	52.94 ± 3.67

Each accuracy is averaged over all subjects and folds. (in 95% confidence level). Bold values represents best results in this set of experiments.

As can be seen in Table 4, there was a statistically significant difference in the performance of different models [Friedman Test $X_{\left(15\right)}^{2}=16.53$, *p* = 0.0002 < 0.05, *Post-hoc* analysis with Wilcoxon signed rank tests was conducted] and our model outperforms EEGNet (*p* = 0.0003 < 0.05) and DeepConv (*p* = 0.005 < 0.05) in most cases through vertical comparison in the table.

Also, there was a statistically significant difference in the performance of different learning structures [$X_{\left(9\right)}^{2}=8.67$, *p* = 0.013 < 0.05] and our learning structure (MTL-10) outperforms the traditional learning structures (TL-10: *p* = 0.0039 < 0.05, ML: *p* = 0.019 < 0.05) by a margin of 0.4–5.4% on accuracy through horizontal comparison and the improvement is much more evident when the data provided by the user for fast adaption (number of shots) is fewer in most cases. Furthermore, DeepConvNet gains much more improvement (about 3–5% in Sch2017) through MTL learning strategy than EEGNet and SPD-CNN net with shallow layers and little parameters. It suggests that the SS operation of MTL can effectively avoid the problem of “catastrophic forgetting” (Lopez-Paz and Ranzato, 2017) (It means forgetting general patterns when adapting to a specific task) and as a result, the performance advantage of large-scale CNN is unleashed thoroughly, especially facing with large-scale data.

Nevertheless, there is no free lunch, DeepConvNet required complex network design, and this kind of large-scale network architecture needs a high level of hardware, which consumes lots of time on the design and calibration in the BCI system. To be specific, the comparison of time complexity and the scale of data of neural networks are shown in Table 5. Table 5 shows that SPD-CNN has a high speed of convergence and shorter training time, which are attributed to the small-scale input data and the plain network structure with little parameter.

**Table 5:** The time complexity and scale of the dataset for different networks are compared in this table.

**Method**	**EEGNet**	**DeepConvNet**	**SPD-CNN**
Dataset	Training Time (minute)		
BNCI2014001	48 m	156 m	**23m**
BNCI2015004	52 m	179 m	**36m**
Sch2017	218 m	418 m	**175m**
	Data Size(Gigabyte)		
BNCI2014001	0.89G		**0.016G**
BNCI2015004	1.5G		**0.022G**
Sch2017	30.8G		**1.7G**

*In this table, we train different network architectures using MTL, then calculate the scale of input data and the average consuming time of all folds. Bold values represents best results in this set of experiments*.

As described above, it can be concluded that the proposed SPD-CNN with few learnable parameters has a stronger feature extraction ability to find an approximate boundary to separate different samples from different labels, when the datasets are well described in the SPD manifold. Moreover, with the improvement coming from the MTL learning structure, the CNN-based model would rapidly adapt to the target domain with efficient usage of target data without forgetting key features learned from the source domain.

## 4. Discussion

### 4.1. Analysis of the SPD descriptor

Extensive experiments above show that the SPD matrices are capable of retaining the discriminative information of brain activity and the information can be effectively extracted by the proposed network.

**Figure 6:**
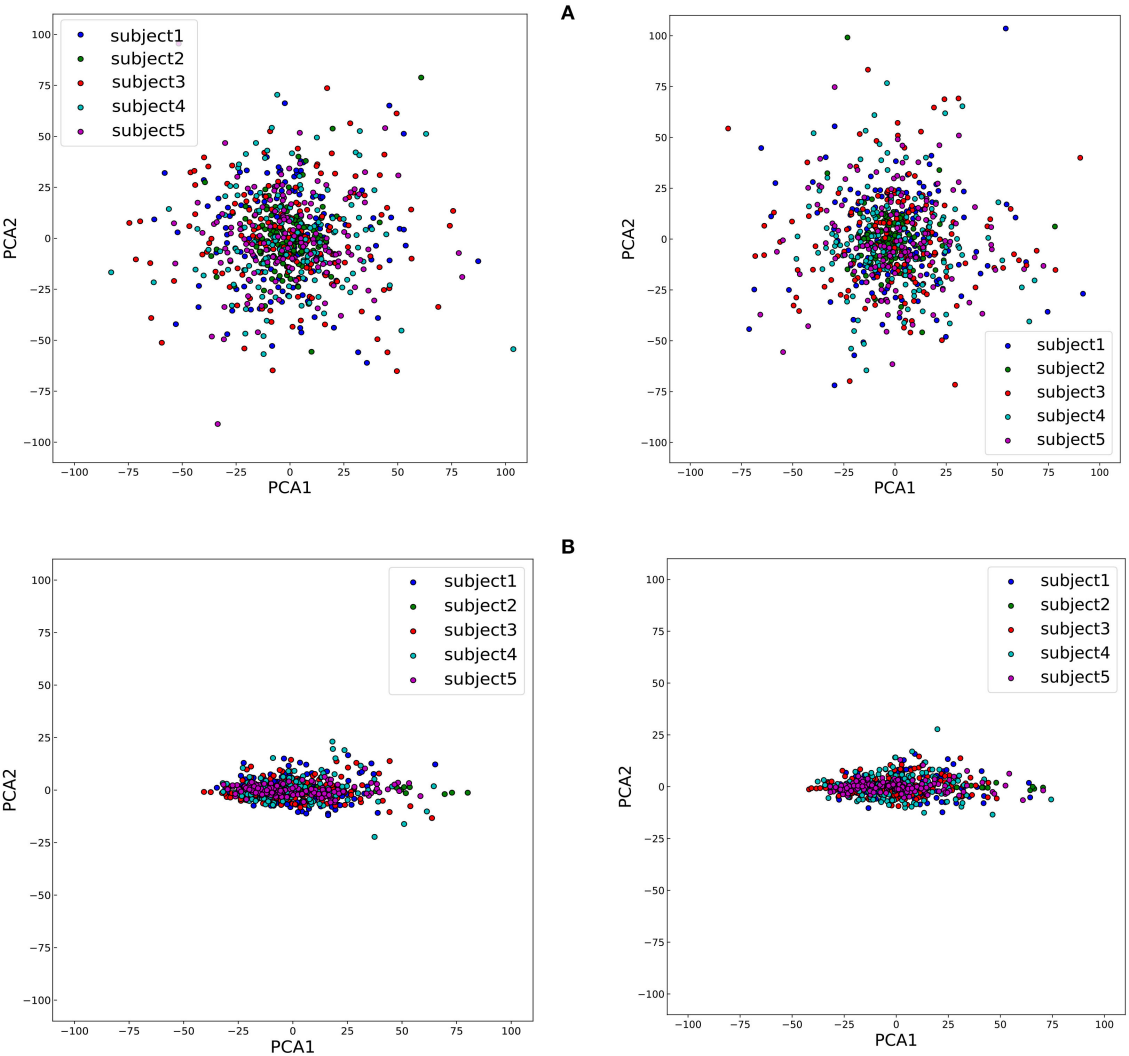
The disparity among five subjects of two classes, right hand and feet, which are on the left and right of the figure, respectively. **(A)** The visualization of sample distributions of raw EEG data. **(B)** The visualization of sample distributions of the SPD matrices.

To study the impact of different data descriptions in the cross-subject learning scenario, the raw EEG data and the SPD matrices of different subjects in BNCI2014001 were reduced to two dimensions by Principal Component Analysis (PCA) and all the samples from the same class were projected to this 2D feature space (Zhang et al., 2018). Consequently, the sample distributions of the different subjects could be visualized in Figure 6. Then we use averaged Euclidean distance to quantitatively measure the distance among different subjects in the feature space, and the result are shown in Table 6.

**Table 6:** The averaged Euclidean distance among different subjects of SPD matrices and EEG data in the feature space.

**Class**	**Euclidean**	**Euclidean**
	**distance**	**distance**
	**(SPD matrices)**	**(EEG data)**
Right hand	8.85	13.85
Feet	8.71	13.72
Left hand	8.90	13.86
Tongue	8.33	13.96

The results of Figure 6 and Table 6 revealed that the gaps in the sample distributions among different subjects were closed by transforming the EEG data into SPD matrices.

### 4.2. Limitations and future directions

Though the proposed network and learning strategy have achieved great performance in the cross-subject scenario, the limitation is still involved in the current study. For the experiments, we only validate our method on the paradigm of motor imagery and the effectiveness of our method on the other paradigm in the EEG-BCI system is still unclear. Therefore, in future studies, we will focus on the other paradigm such as Steadystate Visually Evoked Potential (SSVEP) datasets and further explore the potential of the proposed approach.

## 5. Conclusion

In this study, we represent a brand-new model to extract cognitive information from EEG data. Compared with the two famous EEG networks, which utilize different convolutional layers to learn specific filters, we transform EEG signals into SPD matrices and design a plain CNN to learn the essential features from it. Considering the shifting problem between different subjects, we use the MTL training strategies to train our model and related experiments show that our training strategy is capable of keeping the adaptation ability of the networks while significantly reducing the number of parameters to transfer. It can be concluded that our proposed model performs well in the cross-subject learning scenario.

Our contribution is part of a larger effort in the BCI learning research, intending to design robust algorithms which use the experience of deep learning in image recognition to mitigate inter-subject variability (Xu et al., 2021) and extract shared information between different subjects. Besides, it is easy to notice that we could use more complex CNN-based models, which have the powerful feature extraction ability for SPD data. Given that, the topic considered here also opens several important questions to be investigated in the future. For instance, considering the feasibility of the network to extract the characteristics of the SPD data, to determine how to design the specific network architecture for this kind of data is promising research. Furthermore, with the feature expression based on the SPD form, data formats of different experiments in the same paradigm can be unified, and it allows us to gather information from several databases and use the CNN-based model to form a more robust classifier in the future.

## Data availability statement

The data that support the findings of this study are openly available in https://github.com/NeuroTechX/moabb.

## Ethics statement

Written informed consent was obtained from the individual(s) for the publication of any potentially identifiable images or data included in this article.

## Author contributions

LC developed the theoretical formalism, performed the analytic calculations, and performed the numerical simulations. ZY and JY contributed to the final version of the manuscript. ZY supervised the project. All authors contributed to the article and approved the submitted version.

## Funding

This research was supported in part by the National Natural Science Foundation of China under Grants 61836003 and 61906211 and the Major Program of - Brain Science and Brain-Like Research of the National Science and Technology Innovation 2030 under Grant 2022ZD0211700.

## Conflict of interest

The authors declare that the research was conducted in the absence of any commercial or financial relationships that could be construed as a potential conflict of interest.

## Publisher's note

All claims expressed in this article are solely those of the authors and do not necessarily represent those of their affiliated organizations, or those of the publisher, the editors and the reviewers. Any product that may be evaluated in this article, or claim that may be made by its manufacturer, is not guaranteed or endorsed by the publisher.
